# Supplementary material for: Targeting PKCθ Promotes Satellite Cell Self-Renewal
Source: Int J Mol Sci. 2020 Mar 31;21(7):2419. doi: 10.3390/ijms21072419 (PMC7177808; doi:10.3390/ijms21072419)
Supplement: Supplementary file 1 [file ijms-21-02419-s001.pdf]

Supplementary Materials

Figure S1

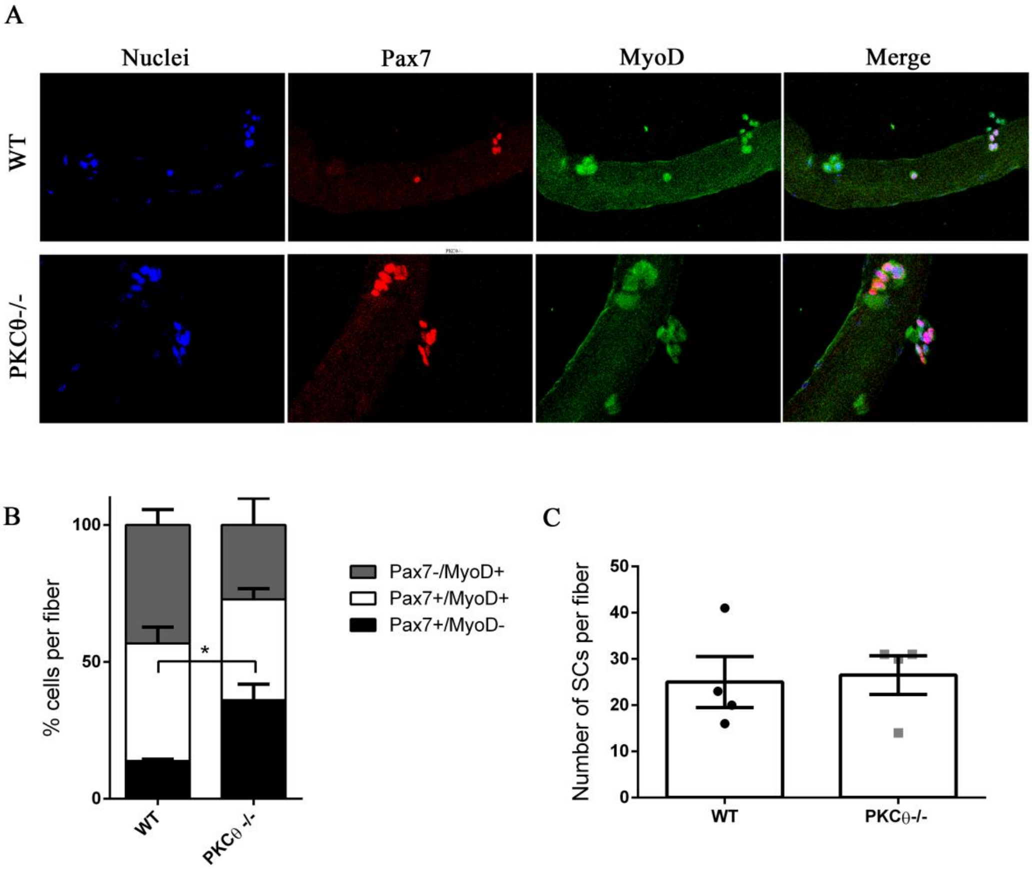

Figure S1: Lack of PKCθ stimulates SC self-renewal *in vitro*. A: Representative pictures of single myofibers isolated from EDL muscles of WT and PKCθ -/- mice. Myofibers were stained for Pax7 (red) and MyoD (green), after 72h of culturing, nuclei were counterstained with Topro3. B: Quantification of SCs per fiber, single or double positive for Pax7 and/or MyoD, after 72h in culture. C: number of SCs per fiber in WT and PKCθ -/- single myofibers, after 72h of culturing. (WT, n = 4 mice, PKCθ-/-, n = 4 mice, n = 100 cells analysed per group). Error bars represent mean ± sem, *$p < 0.05$ calculated by Student's t-test.

Figure S2

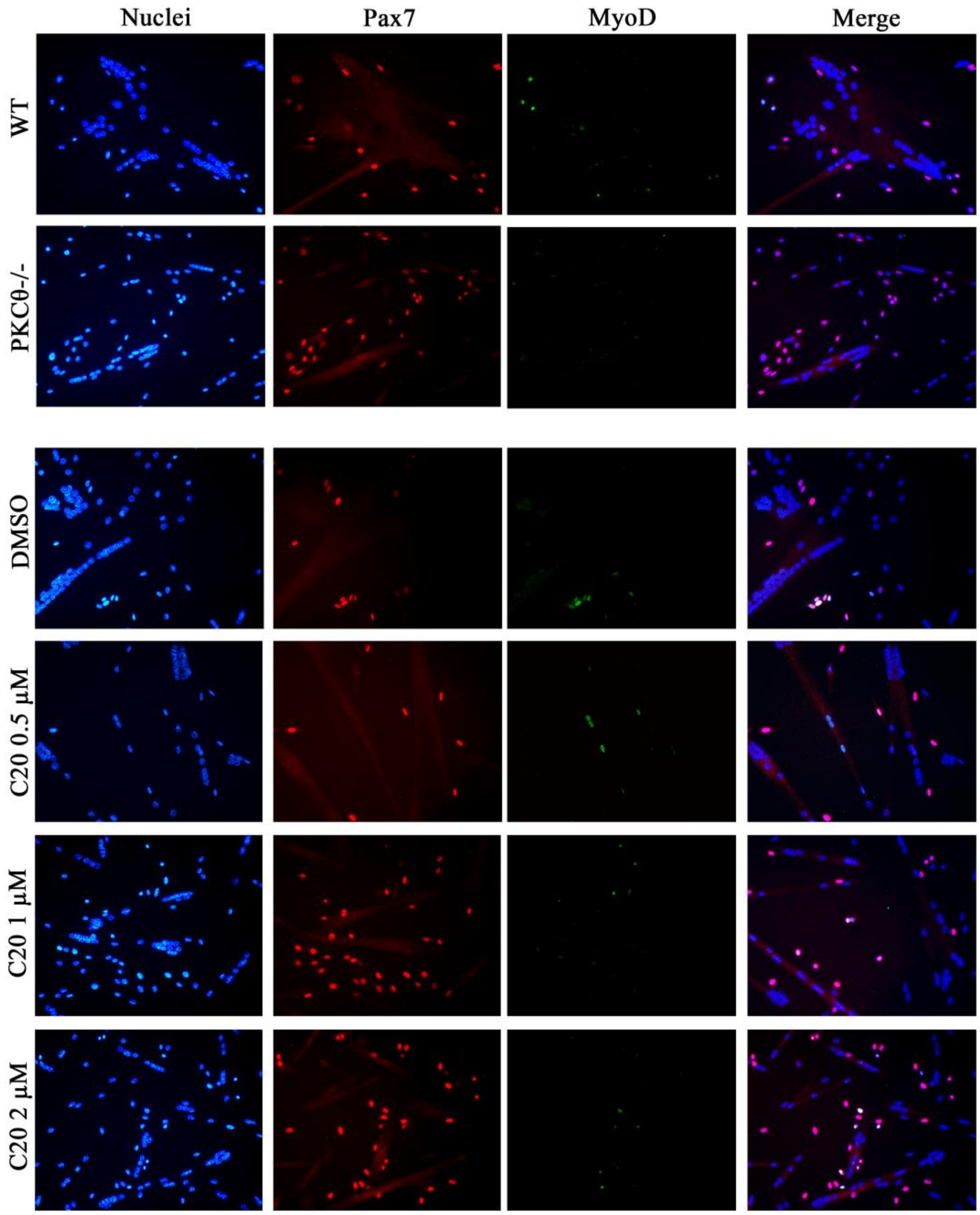

Figure S2: Representative pictures of C20 treated WT and PKCθ-/- SCs. A: Representative pictures of WT and PKCθ-/- SCs, or WT SCs cultured in presence of C20 or Vehicle (DMSO) at the concentration of 0.5, 1 and 2 μM. The cells were stained for Pax7 (red) and MyoD (green) after culturing for 4 days in GM and 2 days in DM. Nuclei were counterstained with Hoechst.

Figure S3

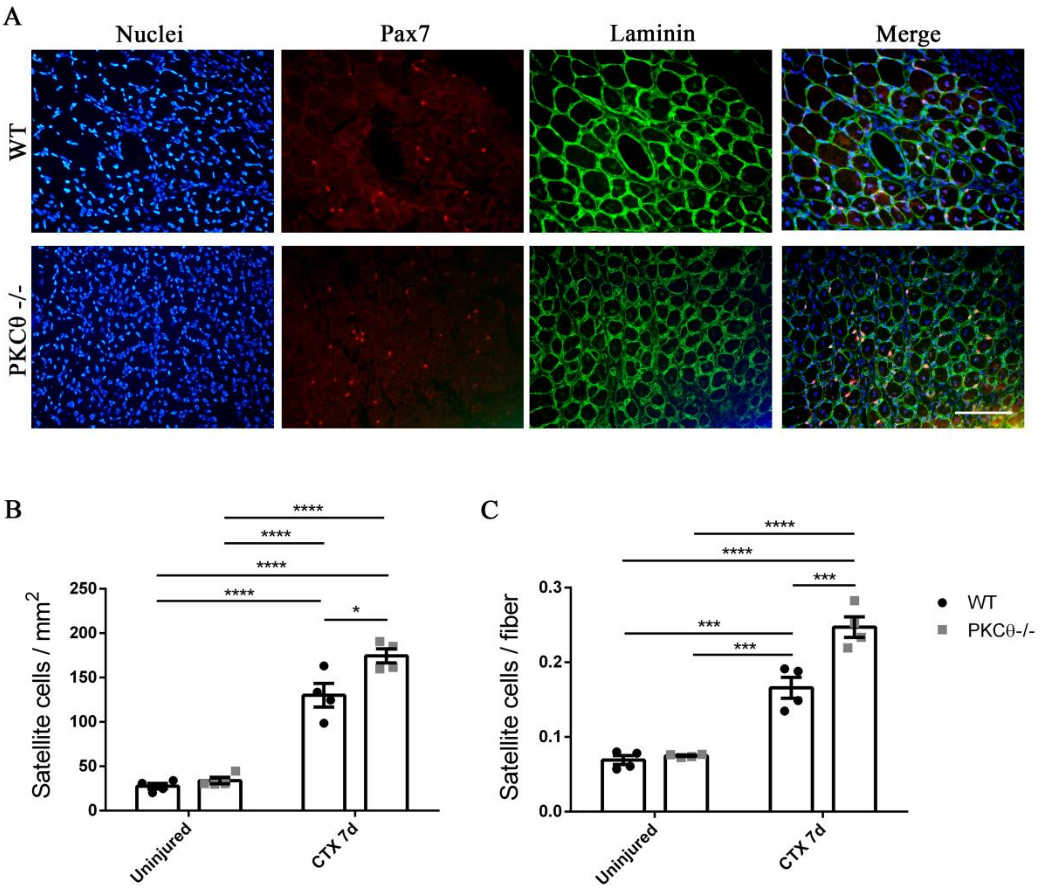

Figure S3: SC number is higher in PKCθ-/- muscles compared to WT, 7 days after injury. A: representative immunofluorescence pictures of WT and PKCθ-/- GA sections, 7 days after CTX injury. Sections were stained for Pax7 (red) and Laminin (green). Nuclei were counterstained with Hoechst. Scale bar: 100μm. B: quantification of the number of SCs per mm^2 and C: number of SCs per fiber in uninjured and 7 day-injured GA muscle, in WT and PKCθ-/- mice (WT, n = 4 mice, PKCθ-/-, n = 4 mice). Error bars represent mean ± sem, *$p < 0.05$, **$p < 0.01$, ***$p < 0.001$, **** $p < 0.0001$ calculated by Two-way Anova with adjustment for multiple comparison test.

Figure S4

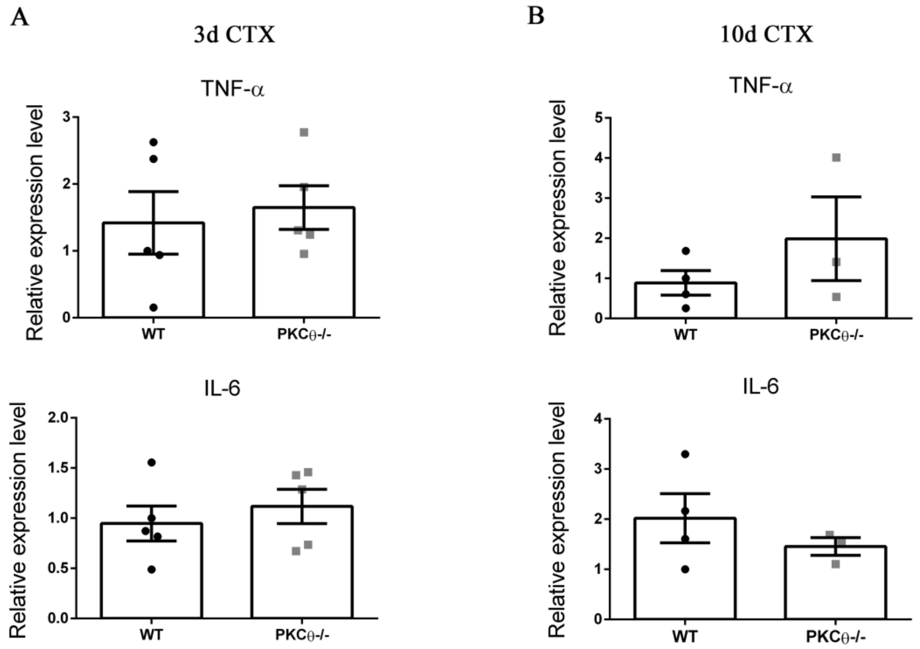

Figure S4: Lack of PKCθ does not affect the inflammatory cytokine expression after CTX injury. A: Real Time PCR analysis of inflammatory cytokines TNF-α, and IL-6 at 3 days after CTX injury and B: 10 days after CTX injury, from total TA muscle RNA extract of WT and PKCθ-/- mice.
